# Comprehensive Analysis of Lung Cancer Metastasis: Sites, Rates, Survival, and Risk Factors—A Systematic Review and Meta‐Analysis

**DOI:** 10.1111/crj.70107

**Published:** 2025-07-11

**Authors:** Shilin Wang, Wen Tang, Fu Jin, Huanli Luo, Han Yang, Ying Wang

**Affiliations:** ^1^ Department of Radiation Oncology Chongqing University Cancer Hospital Chongqing People's Republic of China; ^2^ Department of Rehabilitation The Second Affiliated Hospital of Chongqing Medical University Chongqing People's Republic of China

**Keywords:** lung cancer, meta‐analysis, metastasis, overall survival, risk factors

## Abstract

**Objectives:**

Lung cancer metastasis constitutes a critical aspect of disease progression and patient outcomes. It is imperative to illuminate the complex landscape of lung cancer metastasis, offering a thorough evaluation of sites, rates, risk factors, and survival implications.

**Methods:**

Studies on the prevalence of lung cancer metastasis, the risk factors, the overall survival (OS) after metastasis, or the risk factors for OS were included. Two independent reviewers used a standardized data extraction and quality assessment form. Hazard ratios and confidence intervals were calculated using random‐effects or fixed‐effects models.

**Results:**

This systematic meta‐analysis included 115 clinical trials. Prevalent metastatic sites in non‐small cell lung carcinoma (NSCLC) encompassed brain (29%), bone (25%), adrenal gland (15%), liver (13%), and skin (3%). However, small cell lung carcinoma (SCLC) primarily metastasized to liver (33%), brain (30%), bone (27%), adrenal gland (10%), and pericardium (3%). The risk factors for brain metastases in NSCLC included lung adenocarcinoma, EGFR mutations, and prophylactic cranial irradiation (PCI); in SCLC brain metastasis, age and PCI were influential. The median OS after brain metastasis in NSCLC and SCLC was 21.3 and 10.5 months, respectively, while liver or bone metastases were notably linked to poorer survival. The factors influencing OS in NSCLC brain metastasis included age, EGFR mutations, and stereotactic radiosurgery. For SCLC brain metastasis, OS was notably impacted by gender, smoking status, the number of brain metastases, and radiotherapy.

**Conclusion:**

This study provided insights into lung cancer metastasis. The results revealed the metastatic rates, risk factors, and OS to assist clinical decision‐making.

## Graphical abstract

This meta‐analysis of 115 studies showed common metastatic sites for NSCLC as brain (29%), bone (25%), and adrenal gland (15%); for SCLC, they were liver (33%), brain (30%), and bone (27%). PCI reduced brain metastasis in both cancers. Median OS after brain metastasis was 21.3 months (NSCLC) and 10.5 months (SCLC). Liver and bone metastases worsened survival.

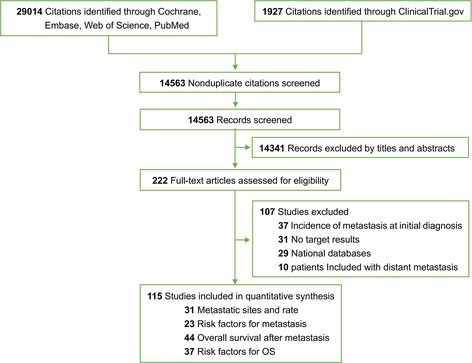

## 1 Introduction

Lung cancer remains a leading cause of cancer‐related mortality worldwide, and its metastatic spread represents a significant challenge in clinical management. Despite advancements in early detection and treatment modalities, lung cancer's propensity to metastasize continues to pose formidable challenges for clinicians and researchers [1]. Recently, a comprehensive analysis of the nationwide Swedish cancer data revealed that overall survival (OS) after diagnosis of metastatic lung cancer was only 4 months for men and 5 months for women—an appreciable diminution when juxtaposed with the more extended survival spans of 12 and 14 months observed in the absence of metastatic dissemination [2].

Unquestionably, metastasis represents a critical turning point in the clinical management of lung cancer patients, as it drastically alters the therapeutic landscape. Localized tumors can often be effectively treated with surgery, radiation, or targeted therapies, but the spread of cancer cells to distant sites would reduce treatment success rates substantially [3]. The intricate phenomenon of lung cancer metastasis entailed a multitude of contributory factors, encompassing the cancer's pathological classification, epithelial growth factor receptor (EGFR) mutations, therapeutic modalities, and more [4, 5, 6]. Meanwhile, non‐small cell lung carcinoma (NSCLC) and small cell lung carcinoma (SCLC) exhibited distinct metastatic tendencies and survival rates, highlighting their heterogeneity [2]. However, prior studies had just underscored the paramount significance of comprehending the intricacies of cancer metastasis patterns and the contributory factors thereto; there was a lack of systematic analysis of metastasis sites, rates, risk factors, and survival implications [7].

Therefore, this systematic meta‐analysis aimed to enhance our understanding of metastatic patterns in NSCLC and SCLC by quantifying distant metastasis rates, identifying risk factors, and revealing survival outcomes. The results may guide future research efforts in identifying new therapeutic targets and refining screening, prevention, and treatment strategies tailored to the specific metastatic profile of each patient.

## 2 Methods

### 2.1 Search Strategy and Selection Criteria

In this systematic review and meta‐analysis, a search was performed in Cochrane, Embase, Web of Science, PubMed databases, and ClinicalTrial.gov (inception to May 8, 2023). We used both subject headings and text‐word terms for “lung neoplasms,” “metastases,” and related and exploded terms, including medical subject headings terms in combination with keyword searching. A full search strategy is presented in the Table S1. This systematic review and meta‐analysis was performed in accordance with a previously published protocol (CRD42023421908). This study followed the Preferred Reporting Items for Systematic Reviews and Meta‐analyses (PRISMA) reporting guideline [8].

### 2.2 Study Selection

After the initial search, we independently screened the titles and abstracts of the articles to identify potentially relevant studies. Subsequently, a full‐text review was performed on studies identified as potentially relevant in the title and abstract review. Finally, we independently screened the full‐text articles to apply inclusion and exclusion criteria. Discrepancies between the reviewers were resolved by discussion. When multiple articles included overlapping series of patients, we preferentially extracted outcome data from the primary article with the largest sample size for early outcomes and from the article with the longest follow‐up duration for late outcomes.

### 2.3 Eligibility Criteria

Randomized controlled trials (RCTs) and cohort studies (CRS) were included in this meta‐analysis if they fell into one of the following three categories: (1) Studies provided metastasis rates of lung cancer to distant organs; (2) studies explored the risk factors for distant metastasis of lung cancer; and (3) studies analyzed the OS rate or risk factors after distant metastasis of lung cancer. However, the following types of studies were excluded: (1) Only the lung cancer metastasis rate at initial diagnosis was provided, but the metastasis rate throughout the clinical process was not reported; (2) all included patients had already experienced distant metastasis; (3) no median survival with 95% confidence interval (CI) was provided when reporting OS. We also excluded review articles, conference abstracts, case reports, studies based on national databases, and unpublished data or gray literature studies.

### 2.4 Quality Assessment

The Newcastle‐Ottawa Scale assesses study bias and assigns points in the following three domains: appropriate selection of participants, appropriate measures of exposure and outcome variables, and appropriate control of confounding [9]. The scale yields a quantitative summary score and qualitative categorization of quality (poor, fair, or good) based on the number of points in the three domains. We independently assessed and scored each study according to the predefined criteria. We also investigated the potential for publication bias by visually inspecting funnel plots for asymmetry and with the Egger regression test [10].

### 2.5 Statistical Analysis

Data were pooled using random‐effects models to allow for significant heterogeneity in the distant metastasis rate. The risk factors for distant metastasis of lung cancer were meta‐analyzed using the fixed effects or random effects model, as appropriate. Heterogeneity was assessed using the *χ* ^2^ test and the *I* ^2^ statistic. Significant heterogeneity was indicated by *p* < 0.05 in Cochrane *Q* tests or a ratio greater than 40% in *I* ^2^ statistics, which led to the use of random‐effects models. Otherwise, these tests were negative for heterogeneity, and fixed‐effects models were chosen. We performed a one‐study‐removed sensitivity analysis, in which the meta‐analysis for each outcome was recalculated after removing one study at a time to determine the association of individual studies with meta‐analysis results. Statistical analyses were performed using the Cochrane Review Manager, Version 5.3, and Stata MP software, Version 13.1. A confidence level of 95% (*p* < 0.05) was considered statistically significant.

## 3 Results

### 3.1 Eligible Studies and Characteristics

**FIGURE 1:**
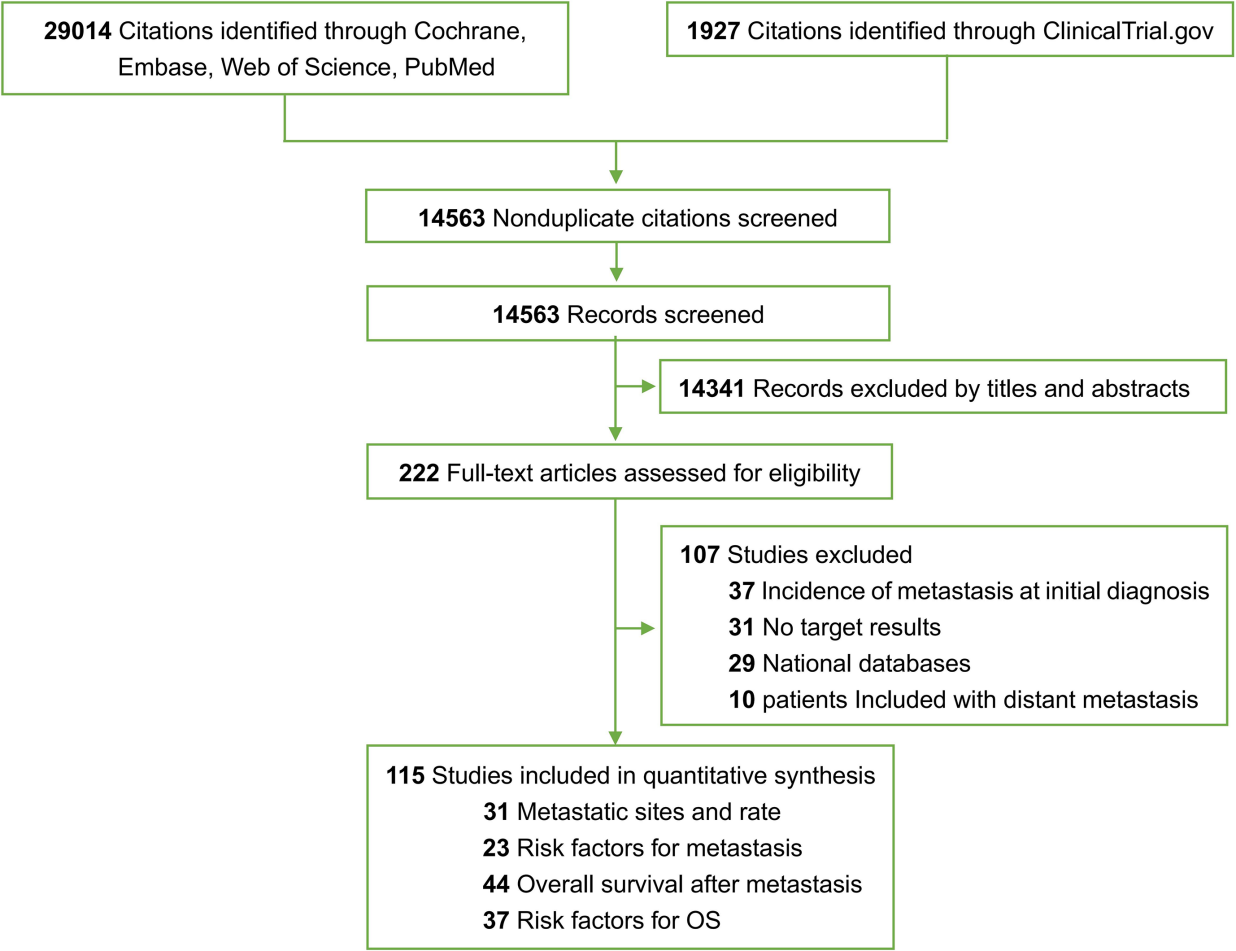
PRISMA flow diagram.

A total of 29 014 publications and 1927 registered clinical studies were identified from the literature search (Table S1). After screening titles and abstracts for eligibility, 222 full‐text articles were reviewed. After further exclusion, 115 articles were eligible for inclusion (Figure 1). Among them, 31 articles were about the incidence of distant metastasis of lung cancer (Table S2) [4, 11, 12, 13, 14, 15, 16, 17, 18, 19, 20, 21, 22, 23, 24, 25, 26, 27, 28, 29, 30, 31, 32, 33, 34, 35, 36, 37, 38, 39, 40], 23 articles were about the risk factors for distant metastasis (Table S3) [5, 6, 12, 16, 20, 41, 42, 43, 44, 45, 46, 47, 48, 49, 50, 51, 52, 53, 54, 55, 56, 57, 58], 44 articles were about the OS after distant metastasis (Table S4) [24, 44, 59, 60, 61, 62, 63, 64, 65, 66, 67, 68, 69, 70, 71, 72, 73, 74, 75, 76, 77, 78, 79, 80, 81, 82, 83, 84, 85, 86, 87, 88, 89, 90, 91, 92, 93, 94, 95, 96, 97, 98, 99, 100], and 37 articles were about the risk factors for OS (Table S5) [44, 47, 60, 65, 73, 76, 77, 80, 85, 89, 90, 91, 96, 99, 100, 101, 102, 103, 104, 105, 106, 107, 108, 109, 110, 111, 112, 113, 114, 115, 116, 117, 118, 119, 120, 121, 122]. Some of the included studies provided relevant data on the incidence of distant metastasis and survival rates after metastasis. Data quality of the remaining articles was assessed using the Newcastle‐Ottawa Scale (Tables S3 and S5).

### 3.2 Metastatic Sites and Rates

**FIGURE 2:**
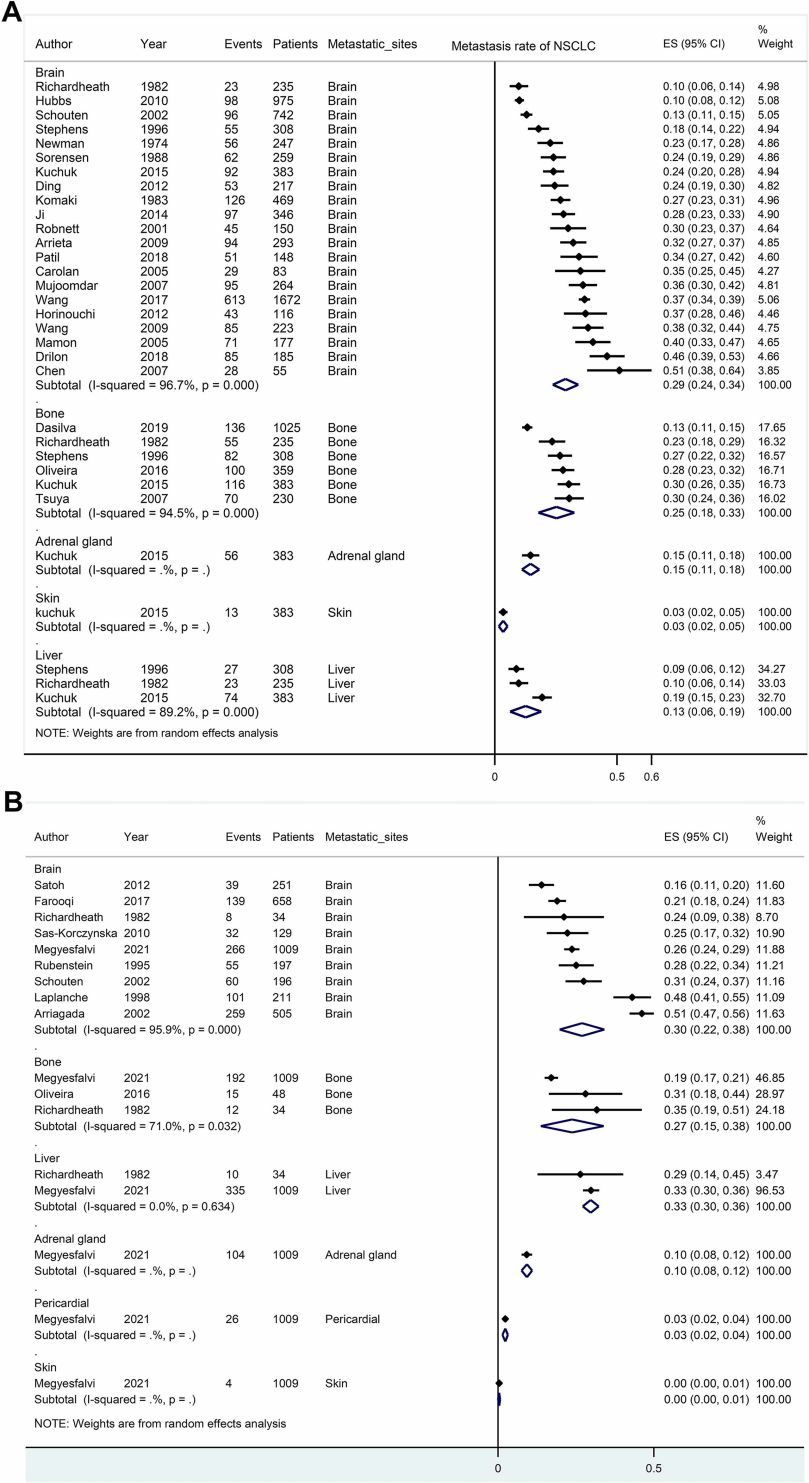
Metastatic sites and rates in lung cancer. (A) NSCLC. (B) SCLC.

Cumulatively, 31 investigations detailed the sites and incidence rates of metastasis in lung cancer. Employing quantitative scrutiny, prevailing metastatic loci in NSCLC encompassed brain (29%), bone (25%), adrenal gland (15%), liver (13%), and skin (3%) (Figure 2A). In the context of SCLC, prominent metastatic sites comprised liver (33%), brain (30%), bone (27%), adrenal gland (10%), and pericardium (3%) (Figure 2B).

### 3.3 Risk Factors for Brain Metastases

Twenty‐three distinct investigations, encompassing a collective cohort of 7173 patients, meticulously examined the risk determinants associated with brain metastases originating from lung cancer. The forest plots disclosed discernible trends: Lung adenocarcinoma exhibited a heightened predisposition to brain metastases within the realm of NSCLC (risk ratio [RR] = 3.59, 95% CI: 1.97–6.54; *p* < 0.001). Furthermore, NSCLC cases bearing EGFR mutations also manifested an elevated propensity for brain metastasis (hazard ratio [HR] = 1.49, 95% CI: 1.14–1.94; *p* = 0.004). Conversely, variables such as sex and smoking did not exert a significant influence on the incidence of brain metastases in the NSCLC context. Intriguingly, the application of prophylactic cranial irradiation (PCI) yielded a substantial reduction in brain metastases incidence among NSCLC patients (HR = 0.36, 95% CI: 0.23–0.56; *p* < 0.001), while chemotherapy exhibited no conspicuous impact on diminishing the rate of brain metastasis (HR = 0.92, 95% CI: 0.55–1.53; *p* = 0.74).

In the context of SCLC, advanced age (≥ 65) (HR = 0.70, 95% CI: 0.54–0.92; *p* = 0.01) exhibited an inverse correlation with brain metastases occurrence. Conversely, variables such as sex and T stage did not exert a significant influence on the incidence of brain metastases within the SCLC cohort. Notably, PCI also demonstrated a substantial capability to reduce the occurrence of brain metastases in SCLC cases (HR = 0.42, 95% CI: 0.30–0.58; *p* < 0.001) (Table 1).

**TABLE 1:** Multivariable predictors for brain metastasis of lung cancer.

Multivariable predictors	No. of trials	No. of patients	Effect size (95% CI)	*p*	*I* ^2^, %
NSCLC					
Adenocarcinoma (yes vs. no)	2	598	RR 3.59 (1.97–6.54)	**< 0.001**	0
Sex (female vs. male)	5	2802	HR 1.15 (0.92–1.45)	0.23	0
Chemotherapy (yes vs. no)	2	700	HR 0.92 (0.55–1.53)	0.74	18
PCI (yes vs. no)	2	496	HR 0.36 (0.23–0.56)	**< 0.001**	0
EGFR mutation (yes vs. no)	4	2763	HR 1.68 (1.35–2.11)	**< 0.001**	34
Smoker (yes vs. no)	2	754	RR 0.81 (0.47–1.40)	0.45	0
SCLC					
Age (≥ 65 vs. < 65)	3	712	HR 0.70 (0.54–0.92)	**0.01**	28
Sex (female vs. male)	3	727	HR 1.17 (0.91–1.51)	0.21	0
T stage (T3‐4 vs. T1‐2)	2	396	HR 2.01 (1.22–3.29)	0.06	0
PCI (yes vs. no)	2	732	HR 0.42 (0.30–0.58)	**< 0.001**	0

*Note:* Bold values denote statistical significance (*p* < 0.05).

### 3.4 Median OS After Brain Metastasis

**FIGURE 3:**
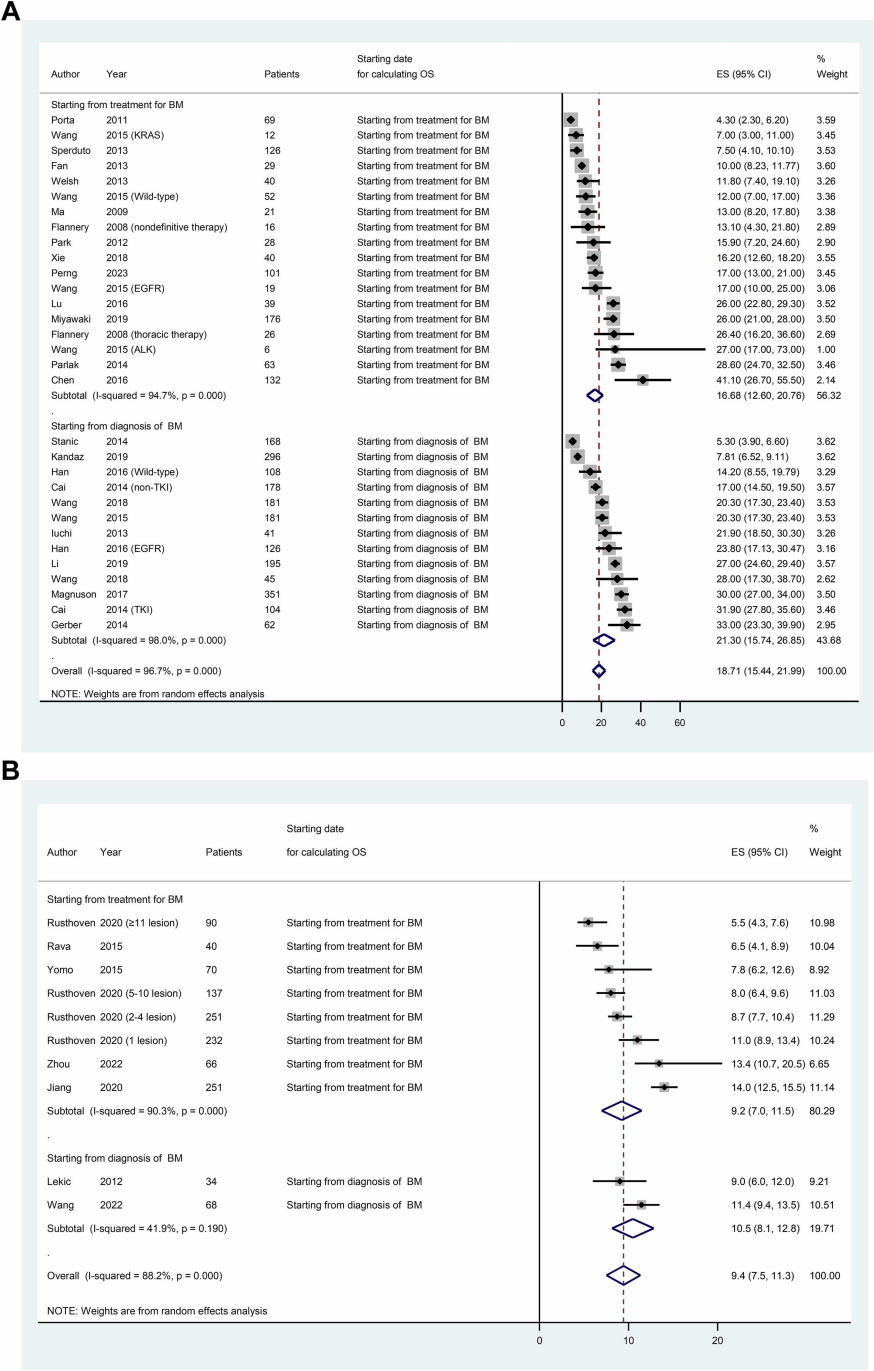
Overall survival after brain metastasis of lung cancer. (A) NSCLC. (B) SCLC. Abbreviation: BM: brain metastasis.

The calculated median OS originating from the commencement of treatment for brain metastasis in NSCLC yielded a duration of 16.7 months (95% CI: 12.6–20.8). Additionally, when measured from the point of brain metastasis diagnosis, the median OS extended to 21.3 months (95% CI: 15.7–26.9; Figure 3A). In the context of SCLC brain metastasis, the median OS arising from treatment initiation amounted to 9.2 months (95% CI: 7.0–11.5). Similarly, calculated from the diagnosis of brain metastasis, the median OS stood at 10.5 months (95% CI: 8.1–12.8; Figure 3B).

### 3.5 Risk Factors for OS After Brain Metastasis

Among elderly individuals afflicted with NSCLC and subsequently encountering brain metastasis, a discernibly diminished OS was observed (HR = 1.90, 95% CI: 1.51–2.39; *p* < 0.001). Notably, patients harboring EGFR mutations experienced an augmented OS (HR = 0.63, 95% CI: 0.52–0.75; *p* < 0.001). Furthermore, the presence of extracranial metastases exerted a substantial negative impact on OS (HR = 2.09, 95% CI: 1.73–2.52; *p* < 0.001). In terms of therapeutic modalities, stereotactic radiosurgery (SRS) demonstrated a noteworthy enhancement of OS when contrasted with tyrosine kinase inhibitor (TKI) treatment (HR = 0.37, 95% CI: 0.26–0.54; *p* < 0.001). Meanwhile, variables such as sex and smoking status evinced no discernible influence on the OS trajectory of NSCLC patients post brain metastasis.

In the context of SCLC brain metastasis, males exhibited a notably shorter OS than their female counterparts (HR = 1.40, 95% CI: 1.22–1.60; *p* < 0.001). Noteworthy, smoking habituated a substantial reduction in OS relative to non‐smoking (HR = 1.33, 95% CI: 1.08–1.65; *p* < 0.008). Moreover, the presence of extracranial metastases was also linked to a discernible diminution in OS (HR = 1.26, 95% CI: 1.01–1.56; *p* < 0.04). It is noteworthy that patients with brain metastases at diagnosis exhibit a higher OS rate compared to those with subsequent metastases (HR = 0.79, 95% CI: 0.67–0.94; *p* < 0.009). A pronounced inverse correlation between the number of brain metastases and survival rate was apparent. Demonstrably effective extracranial control of systemic ailments rendered a significant improvement in survival rates (HR = 2.10, 95% CI: 1.50–2.92; *p* < 0.001). Both whole‐brain radiotherapy (WBRT) with a boost and thoracic radiation therapy emerged as beneficial interventions for augmenting OS (Table 2).

**TABLE 2:** Multivariable predictors for OS of lung cancer.

Multivariable predictors	No. of trials	HR (95% CI)	*p*	*I* ^2^, %
NSCLC				
Age (> 65 vs. ≤ 65)	4	1.90 (1.51, 2.39)	**< 0.001**	0
Sex (male vs. female)	9	0.93 (0.67, 1.28)	0.64	66
Smoking (yes vs. no)	10	1.13 (0.97, 1.31)	0.11	15
EGFR mutation (yes vs. no)	10	0.63 (0.52, 0.75)	**< 0.001**	40
Extracranial metastases (yes vs. no)	7	2.09 (1.73, 2.52)	**< 0.001**	24
SRS vs. TKI	2	0.37 (0.26, 0.54)	**< 0.001**	0
SCLC				
Age (≥ 65 vs. < 65)	3	1.12 (0.85, 1.48)	0.40	10
Age (≥ 60 vs. < 60)	4	1.06 (0.73, 1.55)	0.75	72
Age (older vs. younger)	3	1.01 (1.00,1.02)	0.05	0
Sex (male vs. female)	10	1.40 (1.22, 1.60)	**< 0.001**	0
Smoking (yes vs. no)	5	1.33 (1.08, 1.65)	**0.008**	0
Extracranial metastases (yes vs. no)	3	1.26 (1.01, 1.56)	**0.04**	0
BM symptoms (yes vs. no)	6	1.28 (0.92, 1.79)	0.14	56
BM at diagnosis	2	0.79 (0.67, 0.94)	**0.009**	0
Number of BMs (> 1 vs. ≤ 1)	3	2.32 (1.46, 3.69)	**< 0.001**	0
Number of BMs (> 3 vs. ≤ 3)	3	1.56 (1.01, 2.40)	**0.04**	68
Number of BMs (> 5 vs. ≤ 5)	3	1.27 (1.01, 1.61)	**0.04**	0
Prior WBRT (yes vs. no)	2	0.95 (0.58, 1.55)	0.83	0
Systemic disease extracranial control (uncontrolled vs. controlled)	5	2.10 (1.50, 2.92)	**< 0.001**	0
WBRT plus boost versus WBRT	3	0.64 (0.52, 0.78)	**< 0.001**	0
Thoracic RT (yes vs. no)	4	0.71 (0.57, 0.90)	**0.003**	14

*Note:* Bold values denote statistical significance (*p* < 0.05).

Abbreviation: BM: brain metastasis.

### 3.6 OS Rates for Adrenal, Bone, and Liver Metastases

The median OS computed from the commencement of treatment for lung cancer adrenal metastases exhibited a duration of 13.3 months (95% CI: 10.1–16.5; Figure S1). Furthermore, the median OS stemming from the onset of NSCLC bone metastases was found to be 7.7 months (95% CI: 3.3–12; Figure S2). Correspondingly, for SCLC bone metastases, the calculated median OS from the point of SCLC diagnosis stood at 6.4 months (95% CI: 0.8–12; Figure S3). Notably, adenocarcinoma exhibited heightened susceptibility to bone metastases within the spectrum of lung cancer (HR = 2.31, 95% CI: 0.87–6.13; *p* = 0.04; Figure S4). The median OS calculated from the initiation of NSCLC liver metastases was determined to be 11.2 months (95% CI: 5.7–16.6; Figure S5). Conversely, for SCLC liver metastases, the computed median OS commencing from the date of treatment was a mere 3 months.

### 3.7 Quality Assessment and Publication Bias

Low risk of bias was assessed in several studies. Overall, the risk of bias of the included studies was low and is summarized in Tables S3 and S5. Funnel plot asymmetry was not evident for any outcome (Figure S6), and the results of the Egger regression test did not indicate publication bias (*p* = 0.559, Figure S7).

## 4 Discussion

This study conducted a meta‐analysis of current clinical investigations on lung cancer metastasis. It examined intricate metastasis patterns in NSCLC and SCLC, quantitatively analyzed metastasis rates at various sites during clinical progression, investigated factors influencing metastasis, and assessed OS after metastases.

First of all, this meta‐analysis revealed that metastatic sites mainly encompassed brain, bone, liver, and adrenal gland. It was in line with the findings based on the Swedish Cancer Registry, which highlighted the common sites as the nervous system, bone, liver, and adrenal gland [2]. Meanwhile, they found that nervous system (47%), liver (35%) and bone (25%) metastases were common in SCLC, and bone (32%, 39%), nervous system (30%, 38%), and liver (17%, 17%) metastases were frequent in squamous cell and adenocarcinoma. It revealed marginally elevated metastasis rates in certain sites when compared to the current study. The reason for these discrepancies may be in part due to the fact that the Swedish study only integrated data from both death certificates and the Hospital Discharge Register, while this analysis encapsulated the follow‐up period, accentuating metastasis rates throughout the course of clinical treatment.

Notably, both findings indicated potential variations in site preferences across different histological subtypes, with NSCLC exhibiting a distinct pattern from SCLC. A pronounced distinction between NSCLC and SCLC lay in the larger size of NSCLC cells and their substantially reduced rates of both growth and dissemination, in stark contrast to the aggressive characteristics of SCLC cells [123]. Target organ microenvironment was also thought to be an important factor in metastasis [124]. We hypothesize that the presented differences in metastatic patterns by histological subtypes support the seed‐and‐soil hypothesis. Some histological subtypes (the “seed”) may have a better ability to thrive in target organs (the “soil”) [125]. High propensity of SCLC to metastasize to the liver suggests that the microenvironment of the liver may be more suitable for the survival of SCLC cells with neuroendocrine characteristics compared with NSCLC [126, 127]. On the other hand, the chemokine signaling, adhesion molecules, and growth factors presented in the brain and bone microenvironments create favorable conditions for both NSCLC and SCLC cells to attach, survive, and proliferate, promoting metastatic colonization [128].

Another pivotal facet of this meta‐analysis pertains to the identification of risk factors influencing metastases in lung cancer patients. Our meta‐analysis underscores that metastatic behavior in lung cancer was significantly influenced by four pivotal determinants: age, pathological subtypes, EGFR mutations, and treatment modalities. (1) Brain metastases have indeed been shown to be more common among younger patients. Possible reasons may be that older patients die before brain metastases develop or that metastases are suppressed at other sites in the immunocompetent patient, whereas the brain is an immunoprivileged site [52, 129]. We also acknowledge the fact that younger patients also have a longer survival, therefore increasing the time at risk for developing metastases. (2) Adenocarcinoma cells are prone to infiltrative growth, have strong invasiveness to the vascular wall, and are prone to penetrating the vascular wall and entering the bloodstream [130]. Compared with SCLC, lung adenocarcinoma grows in the periphery of the lung, which has a wide network of small blood vessels and capillaries [131]. Peripheral tumors in the lung are prone to establishing extensive communicating and anastomotic branches with normal blood vessels, resulting in hematogenous metastasis. (3) The EGFR is a transmembrane receptor tyrosine kinase that is involved in various cellular processes. Mutations in the EGFR tyrosine kinase domain occur in approximately 15% of the advanced non‐squamous NSCLC population. EGFR mutation has been confirmed by multiple studies to be an important influencing factor for brain metastasis in NSCLC patients [6, 44, 45, 58]. In the analysis of OS risk factors, EGFR mutations were associated with significantly prolonged OS. However, in real‐world practice, patients with EGFR mutations predominantly receive TKI therapy. Consequently, the observed improvement in OS may be attributable to TKI treatment rather than directly to the EGFR mutations themselves. This interpretation is supported by the established recognition of EGFR mutations as a favorable prognostic factor, linked to enhanced treatment response and improved OS with EGFR‐TKI therapy in multiple randomized trials [132, 133, 134]. However, there is a significantly longer survival for EGFR mutation patients, and therefore a longer period at risk for the development of brain metastases. (4) PCI has a significant effect on reducing brain metastasis in both NSCLC and SCLC. PCI has been explored since the 1970s as a therapeutic option that could lower the rates of brain relapse and possibly increase patients' survival [135].

For the survival rate after metastasis, this meta‐analysis demonstrated that patients with liver or bone metastasis had a shorter survival period. The OS of SCLC with liver metastasis was the shortest, only 3 months. This may be related to the liver being an immunosuppressive organ, thus hampering the immune surveillance of growing metastases in the liver. Liver cancer is also associated with a poor response to chemotherapy [71, 127]. In the context of bone metastasis, around 80% of patients will experience significant pain and reduced quality of life. Moreover, a sharp escalation in the risk of fractures and pain among patients significantly impairs their OS [136].

Finally, the meta‐analysis of risk factors influencing OS subsequent to brain metastasis indicated that advanced age could result in abbreviated OS for individuals experiencing NSCLC brain metastasis. The elderly cancer population, often burdened with comorbidities, was prone to manifesting reduced survival outcomes [137]. Intriguingly, males exhibited diminished OS for SCLC brain metastasis relative to their female counterparts. This phenomenon could potentially be attributed to elevated estrogen levels within the female physiology. Notably, EGFR mutations and SRS collectively conferred an extension of OS in cases of NSCLC brain metastasis. Conversely, factors such as smoking, a higher incidence of intracranial metastases, or the presence of metastases from diverse anatomical sites were associated with curtailed SCLC brain metastasis OS durations. Efficacious interventions such as WBRT with a boost and thoracic radiation therapy extended the capacity to protract OS in SCLC brain metastasis.

This study has several limitations. First, the meta‐analysis of risk factors associated with metastasis rates and OS primarily centers on brain metastasis, given the limited availability of pertinent literature concerning the determinants impacting metastasis across other anatomical sites. Second, although no obvious bias was found by funnel plot and Egger regression test, the majority of included studies were retrospective studies that were prone to inherent bias that could not be detected. Third, the quality of included studies varied to a certain extent, but the results remained robust in the sensitivity analyses. Fourth, the interpretability of our findings is constrained by the predominance of observational cohort studies. While these real‐world studies provide valuable insights into long‐term outcomes, they are inherently more susceptible to confounding factors and selection bias compared to RCTs. We hope that future research will generate additional relevant RCTs to be included in meta‐analyses, enabling more reliable conclusions in subsequent studies.

## 5 Conclusions

This systematic meta‐analysis enhanced understanding of lung cancer metastasis by consolidating diverse data sources, providing comprehensive analysis of sites, rates, risk factors, and survival impact. NSCLC frequently disseminated to the brain and bone, while SCLC predominantly involved the liver, brain, and bone. Metastatic tendencies could be shaped by pathological subtypes, EGFR mutations, and therapeutic interventions. Notably, liver or bone metastases correlated with diminished survival. EGFR mutations and SRS conferred a prolongation in OS for NSCLC brain metastases. Efficacious interventions, such as WBRT with a boost and thoracic radiotherapy, substantively extended the OS of SCLC brain metastases.

## Author Contributions

Mr S.L. Wang and Ms Tang had full access to all the data in the study and take responsibility for the integrity of the data and the accuracy of the data analysis.

*Concept and design:* Dr Jin.

*Acquisition, analysis, or interpretation of data:* S.L. Wang, Tang, and Jin.

*Drafting of the manuscript:* S.L. Wang and Jin.

*Critical revision of the manuscript for important intellectual content:* Jin and Y. Wang.

*Statistical analysis:* S.L. Wang and Tang.

*Administrative, technical, or material support:* Luo and Yang.

*Supervision:* Jin and Y. Wang.

## Conflicts of Interest

The authors declare no conflicts of interest.

## Supporting information


**Data S1** Supplementary Information.

**Table S1** Number of Citations by Each Database and Trial Register Searched.
**Table S2.** Characteristics of included studies on metastasis sites and rates.
**Table S3.** Characteristics of included studies on risk factors for brain metastases.
**Table S4.** Characteristics of included studies on overall survival.
**Table S5.** Characteristics of included studies on risk factors for overall survival.
**Figure S1.** Overall survival after adrenal metastasis.
**Figure S2.** Overall survival after bone metastasis of NSCLC.
**Figure S3.** Overall survival after bone metastasis of SCLC.
**Figure S4.** Risk factors for bone metastasis of lung cancer.
**Figure S5** Overall survival after liver metastasis of NSCLC.
**Figure S6.** Funnel Plots for Main Outcome Comparisons.
**Figure S6A** Funnel Plot of the Meta‐analysis for NSCLC Smoking.
**Figure S6B** Funnel Plot of the Meta‐analysis for SCLC Sex.
**Figure S7.** Egger’s test for Main Outcome.

## Data Availability

The data for this manuscript were extracted from the original manuscripts that have been cited in this work.
