# Supplementary material for: Comprehensive Analysis of Lung Cancer Metastasis: Sites, Rates, Survival, and Risk Factors—A Systematic Review and Meta‐Analysis
Source: Clin Respir J. 2025 Jul 11;19(7):e70107. doi: 10.1111/crj.70107 (PMC12254191; doi:10.1111/crj.70107)
Supplement: Supplementary file 1 — Data S1 Supplementary Information. [file CRJ-19-e70107-s002.docx]

| **Section and Topic** | **Item #** | **Checklist item** | **Location where item is reported** |
| --- | --- | --- | --- |
| **TITLE** | | |  |
| Title | 1 | Identify the report as a systematic review and meta-analysis. | Page 1 (title) |
| **ABSTRACT** | | |  |
| Abstract | 2 | Provide a structured summary including, as applicable: background; objectives; data sources; study appraisal and synthesis methods; results; limitations; conclusions and implications of key findings; systematic review registration number. | Page 4-6 (Abstract) |
| **INTRODUCTION** | | |  |
| Rationale | 3 | Describe the rationale for the review in the context of what is already known. | Page 7, 8 (Introduction) |
| Objectives | 4 | Provide an explicit statement of questions being addressed with reference to participants, interventions, comparisons, outcomes, and study design (PICOS). | Page 7, 8 (Introduction) |
| **METHODS** | | |  |
| Eligibility criteria | 5 | Specify study characteristics (e.g., PICOS) and report characteristics (e.g., years considered) used as criteria for eligibility. | Page 9 (Eligibility Criteria) |
| Information sources | 6 | Describe all information sources (e.g., databases with dates of coverage, contact with study authors to identify additional studies) in the search and date last searched. | Page 8 (Search Strategy and Selection Criteria) |
| Search strategy | 7 | Present full electronic search strategy for at least one database, including any limits used, such that it could be repeated. | Page 8 (Search Strategy and Selection Criteria; Supplementary **eTable 1**) |
| Selection process | 8 | State the process for selecting studies (i.e., screening, eligibility, independently, included in systematic review, and, if applicable, included in the meta-analysis). | Page 8, 9 (Study Selection) |
| Data collection process | 9 | Describe method of data extraction from reports (e.g., independently, in duplicate) and any processes for obtaining and confirming data from investigators. | Page 8, 9 (Study Selection) |
| Data items | 10a | List and define all outcomes for which data were sought. | Page 10 (Statistical Analysis) |
|  | 10b | List and define all variables for which data were sought (e.g., PICOS, funding sources) and any assumptions and simplifications made. | Page 10 (Statistical analysis) |
| Study risk of bias assessment | 11 | Randomized controlled trials were evaluated using the Cochrane Risk of Bias Tool, and cohort studies were analyzed using the Newcastle-Ottawa Scale. | Page 9, 10 (Quality Assessment) |
| Effect measures | 12 | Specify for each outcome the effect measure(s) (e.g. risk ratio, mean difference) used in the synthesis or presentation of results. | Page 10 (Statistical analysis) |
| Synthesis methods | 13a | Describe the processes used to decide which studies were eligible for each synthesis (e.g. tabulating the study intervention characteristics and comparing against the planned groups for each synthesis (item #5)). | Page 10 (Statistical analysis) |
|  | 13b | Describe any methods required to prepare the data for presentation or synthesis, such as handling of missing summary statistics, or data conversions. | Page 10 (Statistical analysis) |
|  | 13c | Describe any methods used to tabulate or visually display results of individual studies and syntheses. | Page 10 (Statistical analysis) |
|  | 13d | Describe any methods used to synthesize results and provide a rationale for the choice(s). If meta-analysis was performed, describe the model(s), method(s) to identify the presence and extent of statistical heterogeneity, and software package(s) used. | Page 10 (Statistical analysis) |
|  | 13e | Describe any methods used to explore possible causes of heterogeneity among study results (e.g. subgroup analysis, meta-regression). | Page 10 (Statistical analysis) |
|  | 13f | Describe any sensitivity analyses conducted to assess robustness of the synthesized results. | Page 10 (Statistical analysis) |
| Reporting bias assessment | 14 | Describe any methods used to assess risk of bias due to missing results in a synthesis (arising from reporting biases). | Page 9, 10 (Quality Assessment) |
| Certainty assessment | 15 | Describe any methods used to assess certainty (or confidence) in the body of evidence for an outcome. | Page 10 (Statistical analysis) |
| **RESULTS** | | |  |
| Study selection | 16a | Describe the results of the search and selection process, from the number of records identified in the search to the number of studies included in the review, ideally using a flow diagram. | Page 11 (Eligible Studies and Characteristics; **Figure 1**) |
|  | 16b | Cite studies that might appear to meet the inclusion criteria, but which were excluded, and explain why they were excluded. | Page 11 (Eligible Studies and Characteristics) |
| Study characteristics | 17 | Cite each included study and present its characteristics. | Page 11 (Eligible Studies and Characteristics; **eTable 2**, **3, 4 and 5**) |
| Risk of bias in studies | 18 | Present assessments of risk of bias for each included study. | Page 15 (Quality Assessment and Publication Bias) |
| Results of individual studies | 19 | For all outcomes, present, for each study: (a) summary statistics for each group (where appropriate) and (b) an effect estimate and its precision (e.g. confidence/credible interval), ideally using structured tables or plots. | **Table 1** and **Table 2** |
| Results of syntheses | 20a | For each synthesis, briefly summarise the characteristics and risk of bias among contributing studies. | Results |
|  | 20b | Present results of all statistical syntheses conducted. If meta-analysis was done, present for each the summary estimate and its precision (e.g. confidence/credible interval) and measures of statistical heterogeneity. If comparing groups, describe the direction of the effect. | Results |
|  | 20c | Present results of all investigations of possible causes of heterogeneity among study results. | **Table 1** and **Table 2** |
|  | 20d | Present results of all sensitivity analyses conducted to assess the robustness of the synthesized results. | Page 15 (Quality Assessment and Publication Bias) |
| Reporting biases | 21 | Present assessments of risk of bias due to missing results (arising from reporting biases) for each synthesis assessed. | Page 15 (Quality Assessment and Publication Bias) |
| Certainty of evidence | 22 | Present assessments of certainty (or confidence) in the body of evidence for each outcome assessed. | Results |
| **DISCUSSION** | | |  |
| Discussion | 23a | Provide a general interpretation of the results in the context of other evidence. | Discussion |
|  | 23b | Discuss any limitations of the evidence included in the review. | Limitations |
|  | 23c | Discuss any limitations of the review processes used. | Limitations |
|  | 23d | Discuss implications of the results for practice, policy, and future research. | Discussion |
| **OTHER INFORMATION** | | |  |
| Registration and protocol | 24a | The review is registered on PROSPERO, number: CRD42023421908. |  |
|  | 24b | https://www.crd.york.ac.uk/prospero/. |  |
|  | 24c | No amendment. |  |
| Support | 25 | This work was partly supported by Chongqing medical scientific research project (Joint project of Chongqing Health Commission and Science and Technology Bureau) (grant no. 2022DBXM005), National Natural Science Foundation of China (grant no. 11575038), and Chongqing Talents Program - Contracted Management System Project (grant no. cstc2021ycjh-bgzxm0023). |  |
| Competing interests | 26 | The authors declare no competing interests. |  |
| Availability of data, code and other materials | 27 | All relevant data is available in this study. |  |

*From:*  Page MJ, McKenzie JE, Bossuyt PM, Boutron I, Hoffmann TC, Mulrow CD, et al. The PRISMA 2020 statement: an updated guideline for reporting systematic reviews. BMJ 2021;372:n71. doi: 10.1136/bmj.n71

For more information, visit: <http://www.prisma-statement.org/>
