# Supplementary material for: Comprehensive Analysis of Lung Cancer Metastasis: Sites, Rates, Survival, and Risk Factors—A Systematic Review and Meta‐Analysis
Source: Clin Respir J. 2025 Jul 11;19(7):e70107. doi: 10.1111/crj.70107 (PMC12254191; doi:10.1111/crj.70107)
Supplement: Supplementary file 2 — Table S1 Number of Citations by Each Database and Trial Register Searched. Table S2. Characteristics of included studies on metastasis sites and rates. Table S3. Characteristics of included studies on risk factors for brain metastases. Table S4. Characteristics of included studies on overall survival. Table S5. Characteristics of included studies on risk factors for overall survival. Figure S1. Overall survival after adrenal metastasis. Figure S2. Overall survival after bone metastasis of NSCLC. Figure S3. Overall survival after bone metastasis of SCLC. Figure S4. Risk factors for bone metastasis of lung cancer. Figure S5 Overall survival after liver metastasis of NSCLC. Figure S6. Funnel Plots for Main Outcome Comparisons. Figure S6A Funnel Plot of the Meta‐analysis for NSCLC Smoking. Figure S6B Funnel Plot of the Meta‐analysis for SCLC Sex. Figure S7. Egger’s test for Main Outcome. [file CRJ-19-e70107-s001.docx]

**Supplementary**

**Supplementary Table 1.** Number of Citations by Each Database and Trial Register Searched

**Supplementary Table 2.** Characteristics of included studies on metastasis sites and rates

**Supplementary Table 3.** Characteristics of included studies on risk factors for brain metastases

**Supplementary Table 4.** Characteristics of included studies on overall survival

**Supplementary Table 5.** Characteristics of included studies on risk factors for overall survival

**Supplementary Figure 1.** Overall survival after adrenal metastasis

**Supplementary Figure 2.** Overall survival after bone metastasis of NSCLC

**Supplementary Figure 3.** Overall survival after bone metastasis of SCLC

**Supplementary Figure 4.** Risk factors for bone metastasis of lung cancer

**Supplementary Figure 5** Overall survival after liver metastasis of NSCLC

**Supplementary Figure 6.** Funnel Plots for Main Outcome Comparisons

**Supplementary Figure 7.** Egger's test for Main Outcome

**Supplementary Table 1.** Number of Citations by Each Database and Trial Register Searched

| **Databases and Trial registers** | **Citations** |
| --- | --- |
| **Databases：** |  |
| Cochrane | 3253 |
| Embase | 8142 |
| Web of Science | 11413 |
| PubMed | 6206 |
| **Total (databases)** | 29014 |
|  |  |
| **Trial registers:** |  |
| USA (ClinicalTrials.gov) | 1927 |

**Cochrane Library databases**

Search Name:

Date Run: 07/05/2023 20:41:03

Comment:

ID Search Hits

#1 ("lung cancer"):ti,ab,kw OR ("lung neoplasms"):ti,ab,kw OR ("non-small cell lung cancer"):ti,ab,kw OR ("small cell lung cancer"):ti,ab,kw (Word variations have been searched) 23378

#2 (metastases):ti,ab,kw OR (metastasis):ti,ab,kw (Word variations have been searched) 29526

#3 #1 AND #2 3253

**Embase session results (10 May 2023)**

No. Query Results

#3 #1 AND #2 8142

#2 metastasis:ti,ab,kw OR metastases:ti,ab,kw 169945

#1 'lung cancer':ti,ab,kw OR 'lung neoplasms':ti,ab,kw OR 'non-small cell lung cancer':ti,ab,kw OR 'small cell lung cancer':ti,ab,kw 175047

**Web of Science**

# Database: All Databases

# Entitlements:

- WOS: 1900 to 2023

- DIIDW: 1966 to 2023

- KJD: 1980 to 2023

- MEDLINE: 1950 to 2023

- PPRN: 1991 to 2023

- PQDT: 1637 to 2023

- SCIELO: 2002 to 2023

# Searches:

1: (((TI=(Lung cancer)) OR TI=(Lung neoplasms)) OR TI=(Non-small cell lung cancer)) OR TI=(Small cell lung cancer) and Preprint Citation Index (Exclude – Database) Date Run: Mon Sep 04 2023 11:50:10 GMT+0800 Results: 225908

2: (TI=(metastasis)) OR TI=(metastases) and Preprint Citation Index (Exclude – Database) Date Run: Mon Sep 04 2023 11:52:18 GMT+0800 Results: 189348

3: #1 AND #2 and Preprint Citation Index (Exclude – Database) Date Run: Mon Sep 04 2023 11:52:23 GMT+0800 Results: 11413

*In order to ensure that the search strategy avoids literature omissions as much as possible, outcome indicators such as cancer metastasis rate, overall survival, risk factors were not included in the search formula.*

**Supplementary Table 2.** Characteristics of included studies on metastasis sites and rates

| **Author** | **year** | **Type** | **Metastatic sites** | **The number of people with metastasis** | **Total number of patients** | **Age (years)** | **Median Follow Up (Months)** | **Country** |
| --- | --- | --- | --- | --- | --- | --- | --- | --- |
| Arriagada | 2002 | SCLC | Brain | 259 | 505 | Mean 57 | NR | France |
| Arrieta | 2009 | NSCLC | Brain | 94 | 293 | Mean ± SE 60.7 ± 0.7 | NR | Mexico |
| Carolan | 2005 | NSCLC | Brain | 29 | 83 | <60: 39 patients, ≥60 44 patients | 24.9 (range 1.8-53.1) | Canada |
| Chen | 2007 | NSCLC | Brain | 28 | 55 | Median 55 (range 38-73) | 37 (range 3-85) | USA |
| Dasilva | 2019 | NSCLC | Bone | 136 | 1025 | Mean 63.4 | NR | Brazil |
| Ding | 2012 | NSCLC | Brain | 53 | 217 | Median 60 (range 27-79) | 71.3 (range 58.7-103.5) | China |
| Drilon | 2018 | NSCLC | Brain | 85 | 185 | Median 60 (range 28-86) | NR | USA |
| Farooqi | 2017 | SCLC | Brain | 139 | 658 | Median 62 (range 27-95) | 21.2 (range 1.2-240.8) | USA |
| Horinouchi | 2012 | NSCLC | Brain | 43 | 116 | NR | NR | Japan |
| Hubbs | 2010 | NSCLC | Brain | 98 | 975 | Median 67 (range 20-93) | 33 (range 1-149) | USA |
| Ji | 2014 | NSCLC | Brain | 97 | 346 | ≤60: 166 patients, >60: 180 patients | 48.3 | China |
| Komaki | 1983 | NSCLC | Brain | 126 | 469 | Adenocarcinoma: median 60.1 (range 34-91), large cell carcinoma: median 62.5 (range 38-88) | NR | USA |
| Kuchuk | 2015 | NSCLC | Brain | 92 | 383 | Median 68（IQR 60-76） | NR | Canada |
| Kuchuk | 2015 | NSCLC | Bone | 116 | 383 | Median 68（IQR 60-76） | NR | Canada |
| Kuchuk | 2015 | NSCLC | Liver | 74 | 383 | Median 68（IQR 60-76） | NR | Canada |
| Kuchuk | 2015 | NSCLC | Adrenal gland | 56 | 383 | Median 68（IQR 60-77） | NR | Canada |
| kuchuk | 2015 | NSCLC | skin | 13 | 383 | Median 68 (IQR 60-76） | NR | Canada |
| Laplanche | 1998 | SCLC | Brain | 101 | 211 | Mean 57.5 | 60 | France |
| Mamon | 2005 | NSCLC | Brain | 71 | 177 | Median 60 (range 35-82) | 34.8 (range 6.5-139.6) | USA |
| Megyesfalvi | 2021 | SCLC | Bone | 192 | 1009 | Median 63 (range 30-91) | 12.6 | Hungary |
| Megyesfalvi | 2021 | SCLC | Liver | 335 | 1009 | Median 63 (range 30-91) | 12.6 | Hungary |
| Megyesfalvi | 2021 | SCLC | Adrenal gland | 104 | 1009 | Median 63 (range 30-91) | 12.6 | Hungary |
| Megyesfalvi | 2021 | SCLC | pericardial | 26 | 1009 | Median 63 (range 30-91) | 12.6 | Hungary |
| Megyesfalvi | 2021 | SCLC | skin | 4 | 1009 | Median 63 (range 30-91) | 12.6 | Hungary |
| Megyesfalvi | 2021 | SCLC | Brain | 266 | 1009 | Median 63 (range 30-91) | 12.6 | Hungary |
| Mujoomdar | 2007 | NSCLC | Brain | 95 | 264 | Median 67 (range 23-99) | NR | Canada |
| Newman | 1974 | NSCLC | Brain | 56 | 247 | Median 54 (range 32-75) | NR | USA |
| Oliveira | 2016 | NSCLC | Bone | 100 | 359 | Mean 63.4 (range 32–87) | >24 | Brazil |
| Oliveira | 2016 | SCLC | Bone | 15 | 48 | Mean 63.4 (range 32–87) | >24 | Brazil |
| Patil | 2018 | NSCLC | Brain | 51 | 148 | Median 61 (range 22-96) | ROS1-positive: 30, ALK-positive: 47 | USA |
| Richardheath | 1982 | NSCLC | Brain | 23 | 235 | Mean 64 | NR | USA |
| Richardheath | 1982 | SCLC | Brain | 8 | 34 | Mean 64 | NR | USA |
| Richardheath | 1982 | NSCLC | Bone | 55 | 235 | Mean 64 | NR | USA |
| Richardheath | 1982 | SCLC | Bone | 12 | 34 | Mean 64 | NR | USA |
| Richardheath | 1982 | NSCLC | Liver | 23 | 235 | Mean 64 | NR | USA |
| Richardheath | 1982 | SCLC | Liver | 10 | 34 | Mean 64 | NR | USA |
| Robnett | 2001 | NSCLC | Brain | 45 | 150 | Median 63 (range 31-77) | 15 (range 1.5-58) | USA |
| Rubenstein | 1995 | SCLC | Brain | 55 | 197 | Mean 66 (range 33-86) | 36.4 (range 4.7-89.8) | USA |
| Sas-Korczynska | 2010 | SCLC | Brain | 32 | 129 | Median 56 (range 33-73) | 19 (range 4-135) | Poland |
| Satoh | 2012 | SCLC | Brain | 39 | 251 | Median 71 (range 41-86) | NR | Japan |
| Schouten | 2002 | NSCLC | Brain | 96 | 742 | NR | NR | USA |
| Schouten | 2002 | SCLC | Brain | 60 | 196 | NR | NR | Netherland |
| Sorensen | 1988 | NSCLC | Brain | 62 | 259 | Median 57 (range 31-70) | 38.1 (range 23.7-65.2) | Denmark |
| Stephens | 1996 | NSCLC | Brain | 55 | 308 | Median 62 (range 37-77) | 30 (range 7-84) | UK |
| Stephens | 1996 | NSCLC | Bone | 82 | 308 | Median 62 (range 37-77) | 31 (range 7-84) | UK |
| Stephens | 1996 | NSCLC | Liver | 27 | 308 | Median 62 (range 37-77) | 32 (range 7-84) | UK |
| Tsuya | 2007 | NSCLC | Bone | 70 | 230 | Median 65 | NR | Japan |
| Wang | 2017 | NSCLC | Brain | 613 | 1672 | Median 56 (range 18-82) | NR | China |
| Wang | 2009 | NSCLC | Brain | 85 | 223 | Median 56 (range 24-77) | NR | China |

**Supplementary Table 3.** Characteristics of included studies on risk factors for brain metastases

| **Author** | **Year** | **Type** | **Effect size** | **Total Sample Size** | **Age in Years** | **Median Follow-Up (Months)** | **Country** | **Newcastle-Ottawa Scale** |
| --- | --- | --- | --- | --- | --- | --- | --- | --- |
| Arrieta | 2009 | NSCLC | RR | 293 | Mean ±SE 60.7 ± 0.7 | NR | Mexico | 6 |
| Bajard | 2004 | NSCLC | RR | 305 | Median 62 (range 33-88) | NR | France | 7 |
| Chang | 2018 | NSCLC | HR | 491 | Mean 62.8 | NR | China | 5 |
| Ding | 2012 | NSCLC | RR | 217 | Median 60 (range 27–79) | 71.3 (range 58.7–103.5) | China | 9 |
| Han | 2016 | NSCLC | HR | 234 | Median 57.5 (range 27–87) | 16.2 (range 1.0-94.4) | China | 6 |
| Horinouchi | 2012 | NSCLC | HR | 116 | Median 57 (range 35-74) | NR | Japan | 6 |
| Hsu | 2016 | NSCLC | HR | 543 | Median 66 (range 30-91) | 34.9 | Canada | 8 |
| Kim | 2021 | NSCLC | HR | 1495 | Mean ± SD 65 ± 10 | 17.9 (range 0.1–31.1) | Korea | 8 |
| Lee | 2021 | NSCLC | OR | 270 | Median 71 (IQR 63–77) | 27.35 (95% CI: 24.99–29.71) | Korea | 7 |
| Li | 2014 | NSCLC | HR | 156 | PCI: median 55 (range 31–73), no-PCI: 57 (range 24-75) | 68.1 (range, 1.1-97.3) | China | 9 |
| Lucaceresoli | 2002 | NSCLC | OR | 112 | Median 58 (range 37–72) | 63 (range 36–120) | Italy | 7 |
| Ouyang | 2020 | NSCLC | HR | 157 | BM: median 54 (range 33–75), no-BM: median 60 (range 28–93) | 24.1 (95%CI: 19.6–28.6) | China | 7 |
| Sun | 2019 | NSCLC | HR | 340 | Mean 61 | 25.2 | Canada | 8 |
| Yang | 2018 | NSCLC | RR | 598 | Median 61 (IQR 55–69) | NR | Korea | 7 |
| Bang | 2017 | SCLC | HR | 397 | Median 66 (range 43-89) | >36 | Canada | 8 |
| Chen | 2016 | SCLC | HR | 204 | Median 58 (IQR 52–63) | 11.2(range 2.9-71.7) | China | 7 |
| Gregor | 1997 | SCLC | HR | 335 | Median 61 (range 33-75) | 43 | Europe | 9 |
| Kim | 2019 | SCLC | HR | 234 | Median 61 (range 34-77) | 22 (range 1-150) | Korea | 7 |
| Sahmoun | 2005 | SCLC | HR | 230 | Median 67 (range 41-89) | NR | USA | 5 |
| Suzuki | 2018 | SCLC | HR | 293 | Median 64 (IQR 58–71) | 14.3 (IQR 9.3–22.8) | USA | 7 |
| Zheng | 2018 | SCLC | HR | 153 | Median 59 (range 23-80) | 42.5 (range 5.8-93.2) | China | 7 |

**Supplementary Table 4.** Characteristics of included studies on overall survival

| **Author** | **Year** | **Type** | **Metastatic sites** | **Sample size** | **Age (years)** | **Follow-Up (Months)** | **Median OS** | **Country** |
| --- | --- | --- | --- | --- | --- | --- | --- | --- |
| Cai | 2014 | NSCLC | Brain | 282 | Median 65 (range 30–78) | Median 28 (range 22-34) | 31.9 vs 17.0 months (TKI vs none) from diagnosis | China |
| Castañón | 2015 | NSCLC | Liver | 40 | NR | NR | 10 (95% CI 2.8-17.2) months after LM | Spain |
| Chen | 2016 | NSCLC | Brain | 132 | EGFR-TKI: 52 (range 29–75), EGFR-TKI plus  WBRT: 52 (range 31–74) | Median 36.2 | 41.1 (95% CI 26.7-55.5) months from treatment | China |
| Fan | 2013 | NSCLC | Brain | 29 | Median 55 (range 23–75) | Median 12.5 (range 1–49) | 10 (95% CI 8.2-11.8) months from treatment | China |
| Flannery | 2008 | NSCLC | Brain | 42 | Median 58 (range 38–74) | Median 64.5 (range 9–150) | 18 (range1.5–150) months, thoracic therapy: 26.4 (95% CI 16.2–36.6), nondefinitive therapy: 13.1 (95% CI 4.3–21.8) from the date of NSCLC diagnosis | USA |
| Gerber | 2014 | NSCLC | Brain | 62 | Mean ± SD Erlotinib: 62 ± 13, WBRT: 58 ± 11, SRS: 61 ± 11 | Median 20 (range 3-75) | 33 (95% CI 23.3-39.9) months from diagnosis of BM | USA |
| Han | 2016 | NSCLC | Brain | 234 | Median 57.5 (range 27–87) | Median 16.2 (range 1.0-94.4) | EGFR-mutant: 23.8 (95%  CI 17.13-30.47), wild-type EGFR: 14.2 (95% CI 8.55-19.79) | China |
| Kandaz | 2019 | NSCLC | Brain | 296 | Median 60.8 (range 21-85) | Median 7 (range 1–57) | 7.81 (95% CI 6.52–9.11) months after diagnosis | Turkey |
| Kitadai | 2020 | NSCLC | Liver | 41 | Median 68 (range 46–83) | NR | 3.12 (95% CI 1.71–9.03) months from the first day of treatment | Japan |
| Lekic | 2012 | SCLC | Brain | 34 | Median 65 | NR | 9 (95% CI 6-12) months from the date of diagnosis | Slovenia |
| Li | 2019 | NSCLC | Brain | 195 | Median (range) WBRT first: 59 (31–71), TKI+WBRT: 57 (34–74), TKI first: 58 (36–76) | Median 27 (range 1-72) | 27 (95% CI 24.6-29.4) months from the date of diagnosis | China |
| Lu | 2016 | NSCLC | Brain | 39 | Median 56 (range 39–73) | Median 25 (range 7-55) | 26 (95% CI, 22.8–29.3) months from starting EGFR TKIs treatment | China |
| Ma | 2009 | NSCLC | Brain | 21 | Median 61 (range 37-74) | Median 15 (range 1-23) | 13 (95% CI 8.2–17.8) months from the beginning of treatment | China |
| Magnuson | 2017 | NSCLC | Brain | 351 | Median (interquartile range) EGFR-TKI: 60 (53-70), WBRT: 58 (51-65), SRS: 63 (54-70) | Median 22 (interquartile range 13-35) | 30 (95% CI 27-34) months from diagnosis | USA |
| Miyawaki | 2019 | NSCLC | Brain | 176 | Median (range) TKI: 67 (41-88), LT: 69 (32-85) | Median 23 (range 2.3-91) | 26 (95% CI 21-28) months from treatment | Japan |
| Parlak | 2014 | NSCLC | Brain | 63 | Median 58 (range 40-69) | Median 25.3 (range 7.1-52.1) | 28.6 (95% CI 24.7-32.5) months from the first day of BM treatment | Turkey |
| Perng | 2022 | NSCLC | Brain | 101 | <60: 43 patients, ≥60: 58 patients | NR | 17 (95% CI 13-21) months from the brain surgery | China |
| Rava | 2015 | SCLC | Brain | 40 | Median 61 (range 36-79) | Median 4 | 6.5 months from treatment | USA |
| Rinaldi | 2019 | NSCLC | Bone | 264 | Median 72 (range32–93) | NR | 11.4 (95% CI 9.4–13.4) months from beginning of first-line treatment for NSCLC | Italy |
| Rusthoven | 2020 | SCLC | Brain | 710 | Median 68.5 (IQR range 62-74) | NR | 8.5 (95% CI 7.9-9.5) months after SRS | USA |
| Sperduto | 2013 | NSCLC | Brain | 126 | Median arm 1: 64, arm 2: 63, arm 3: 61 | Median 33.6 | 7.5 (95% CI 4.1-10.1) months from treatment | USA |
| Stanic | 2014 | NSCLC | Brain | 168 | Median 64 (range 25−88) | NR | 5.3 (95% CI 3.9–6.6) months, EGFR positive: 6.3 months, EGFR negative: 4.8 months | Slovenia |
| Wang | 2018 | NSCLC | Brain | 181 | Median 59 (range 31-77) | Median 16.8 (range 3.6-63.4) | 20.3 (95% CI 17.3–23.4) months from the day of diagnosis NSCLC with brain metastases | China |
| Wang | 2022 | SCLC | Brain | 68 | NR | Median 40 | 11.43 (95% CI 9.39–13.48) months from diagnosis | China |
| Yomo | 2014 | SCLC | Brain | 41 | Median 69 | Median 8.1 (range 0.8-37.8) | 7.8 (95% CI 6.2-12.6) months from the date of initial SRS treatment | Japan |
| Zhao | 2022 | NSCLC | Liver | 23 | Median 55.2 ± 10.7 | NR | 22 (95% CI 16.8–27.2) months after the initiation of CSM-TACE | China |
| Zhou | 2022 | SCLC | Brain | 66 | <60: 42 patients, ≥60: 24 patients | Median >5.7 | 13.4 (95% CI 10.7–20.5) months from the start of treatment | China |

**Supplementary Table 5.** Characteristics of included studies on risk factors for overall survival

| **Author** | **Year** | **Cancer type** | **Sample size** | **Age (years)** | **Median Follow-Up (Months)** | **Country** | **Newcastle-Ottawa Scale** |
| --- | --- | --- | --- | --- | --- | --- | --- |
| Byeon | 2016 | NSCLC | 573 | Median 60 (range 30–86) | 18.4 (range 0.4-47.9) | Korea | 6 |
| Cai | 2014 | NSCLC | 282 | Median 65 (range 30–78) | 28 (range 22-34) | China | 7 |
| Chang | 2018 | NSCLC | 491 | ≥60: 293 patients, <60: 198 patients | NR | China | 5 |
| Doherty | 2017 | NSCLC | 184 | Median 59 (range 29–86) | NR | Canada | 6 |
| Eichler | 2010 | NSCLC | 93 | Mean 60.9+11 | NR | USA | 7 |
| Fan | 2013 | NSCLC | 210 | Median 55 (range 23–73) | 12.5 (range 1–49) | China | 6 |
| Gerber | 2014 | NSCLC | 110 | Mean ± SD Erlotinib: 62 ± 13, WBRT: 58 ± 11, SRS: 61 ± 11 | 20 (range 3-75) | USA | 8 |
| Han | 2016 | NSCLC | 234 | Median 57.5 (range 27–87) | Median 16.2 (range 1.0-94.4) | China | 6 |
| He | 2021 | NSCLC | 73 | Median 57 (range 30–75) | Median 8 | China | 6 |
| Hsiao | 2013 | NSCLC | 505 | ≥60: 81 patients, <60: 58 patients | Median 14.8 (IQR = 7.8–25.8) | China | 7 |
| Hyun | 2020 | NSCLC | 173 | <65: 112 patients, ≥65: 61 patients | Median 18.7 (range 1.6–76.8) | Korea | 6 |
| Jünger | 2021 | NSCLC | 216 | Median 62 (range 38–87) | Mean 8 (range 1–79) | Germany | 7 |
| Li | 2019 | NSCLC | 195 | Median (range) WBRT first: 59 (31–71), TKI+WBRT: 57 (34–74), TKI first: 58 (36–76) | Median 27 (range 1-72) | China | 6 |
| Magnuson | 2017 | NSCLC | 351 | Median (interquartile range) EGFR-TKI: 60 (53-70), WBRT: 58 (51-65), SRS: 63 (54-70) | Median 22 (interquartile range 13-35) | USA | 9 |
| Miyawaki | 2019 | NSCLC | 176 | Median (range) TKI: 67 (41-88), LT: 69 (32-85) | Median 23 (range 2.3-91) | Japan | 8 |
| Perng | 2022 | NSCLC | 101 | <60: 43 patients, ≥60: 58 patients | NR | China | 6 |
| Stanic | 2014 | NSCLC | 168 | Median 64 (range 25-88) | NR | Slovenia | 5 |
| Wang | 2015 | NSCLC | 89 | 63 (range 35-89) | Median 12 | USA | 8 |
| Wang | 2018 | NSCLC | 181 | Median 59 (range 31-77) | Median 16.8 (range 3.6-63.4) | China | 7 |
| Yang | 2017 | NSCLC | 147 | Median 60 (range: 32–90) | Median 13.5 (range: 2.2–67.1) | China | 7 |
| Zhuang | 2013 | NSCLC | 54 | Median WBRT 63 (range 43-81); WBRT + erlotinib 60 (range 37-76) | NR | China | 5 |
| Bernhardt | 2016 | SCLC | 76 | Median 44–60: 41 patients, 61–70: 26 patients, >70: 9 patients | NR | France | 6 |
| Chuang | 2020 | SCLC | 190 | PCI: Median 66 (range, 47–87), observation: 70 (range, 42–89) | Median 10.6 months (range, 1.5–56.2 months) | Korea | 7 |
| Cordeiro | 2019 | SCLC | 41 | Median 59 (range 38-87) | Median 6 (0-43) months | USA | 6 |
| Harris | 2012 | SCLC | 51 | PCI: Median 64 (50–73), Therapeutic WBI 60 (38–73) | NR | USA | 6 |
| Jiang | 2020 | SCLC | 251 | ≤40: 16 patients, 41–50: 52 patients, 51–60: 90 patients, 61–70: 66 patients, ≥71: 27 patients | NR | China | 5 |
| Li | 2021 | SCLC | 180 | Mean 60 ± 8 years (range: 34–87) | Media 40 (range, 1–96 months). | China | 6 |
| Ma | 2022 | SCLC | 21 | Median 61 (range 37-74) | Median 15 (range 1-23) | China | 7 |
| Ni | 2020 | SCLC | 263 | Median 61 | Median 10.1 | China | 7 |
| Rava | 2015 | SCLC | 40 | Median 61 (range 36-79) | Median 4 | USA | 6 |
| Rusthoven | 2020 | SCLC | 710 | Median 68.5 (IQR range 62-74) | NR | USA | 7 |
| Sun | 2018 | SCLC | 82 | Median 59 (range 39–73) | Median 11.4 (range 0.3–95.4) | China | 7 |
| Wang | 2022 | SCLC | 68 | median 54 | Median 40 | China | 6 |
| Yomo | 2014 | SCLC | 41 | Median 69 | Median 8.1 (range 0.8-37.8) | Japan | 7 |
| Zheng | 2023 | SCLC | 49 | Median 64 (range 45–75) | Median 12 months (range, 5–54 months | China | 7 |
| Zhou | 2022 | SCLC | 66 | <60: 42 patients, ≥60: 24 patients | Median >5.7 | China | 6 |
| Zhuang | 2020 | SCLC | 250 | Median 59 (range 33-79) | Median 18.9 (range, 0.5-207.3) | China | 6 |

**Supplementary Figure 1.** Overall survival after adrenal metastasis

*LC：lung cancer；SBRT：stereotactic body radiation therapy.*

**Supplementary Figure 2.** Overall survival after Bone metastasis of NSCLC

**Supplementary Figure 3.** Overall survival after Bone metastasis of SCLC

**Supplementary Figure 4.** Risk factors for bone metastasis of lung cancer


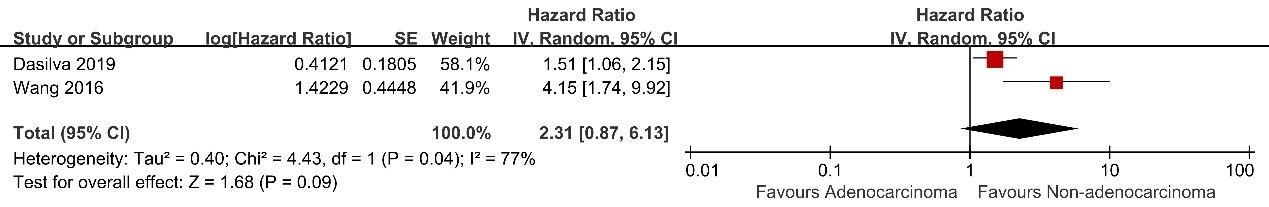


**Supplementary Figure 5.** Overall survival after liver metastasis of NSCLC

*LM：Liver Metastasis.*

**Supplementary Figure 6.** Funnel Plots for Main Outcome Comparisons (Number of studies ≥ 10)

*
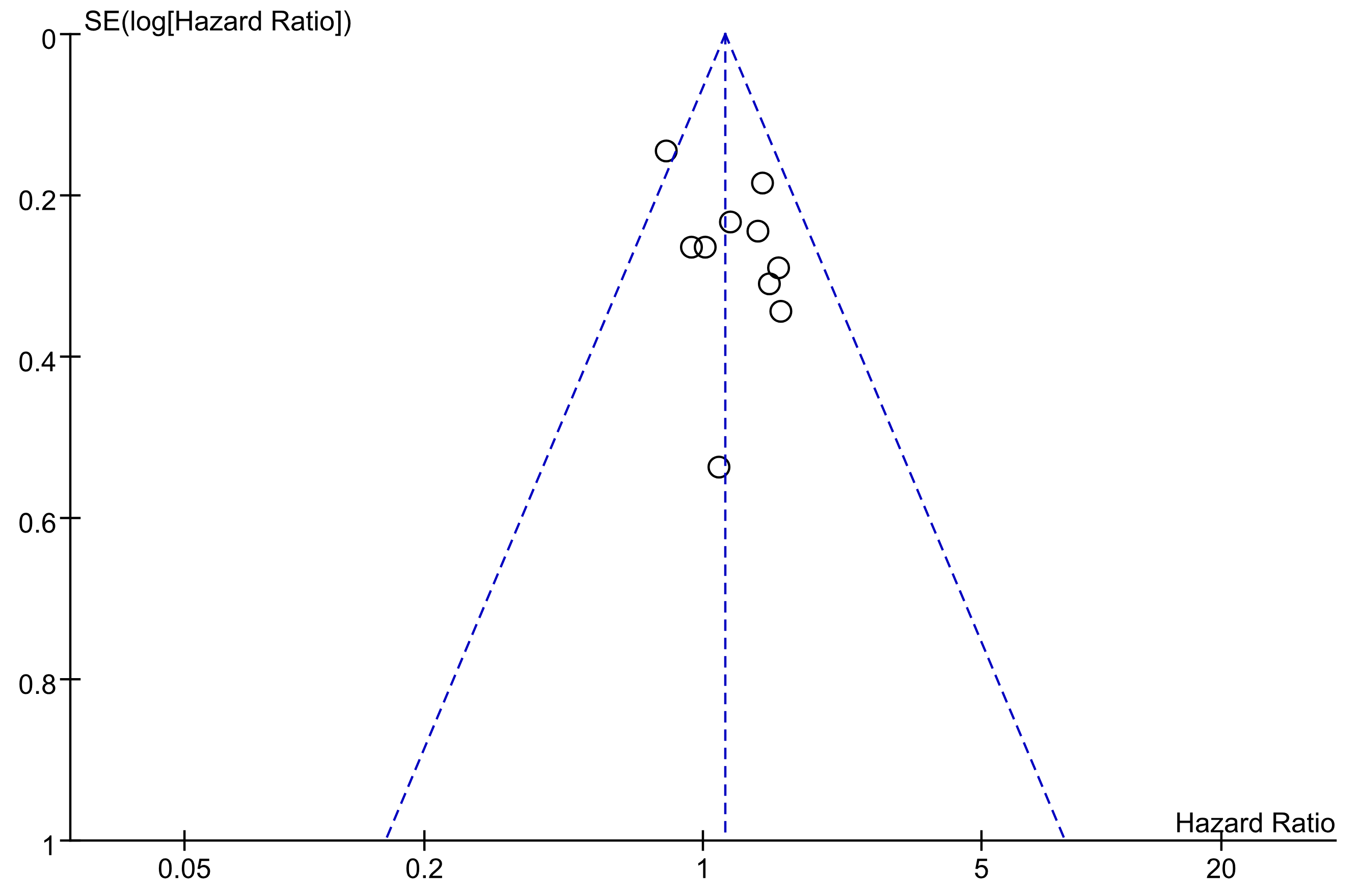
*

**Supplementary Fig. 6A** Funnel Plot of the Meta-analysis for NSCLC Smoking

*
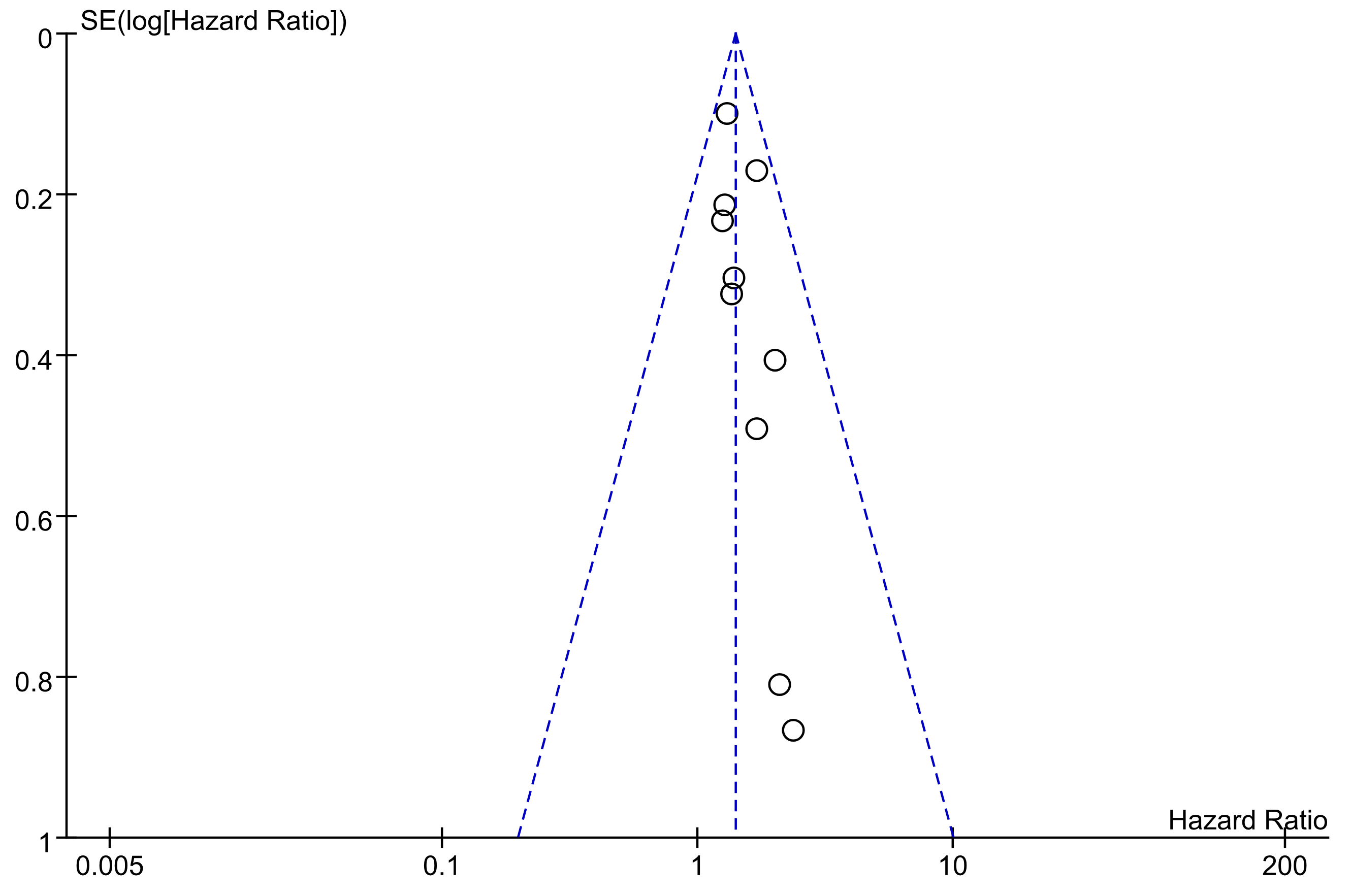
*

**Supplementary Fig. 6B** Funnel Plot of the Meta-analysis for SCLC Sex


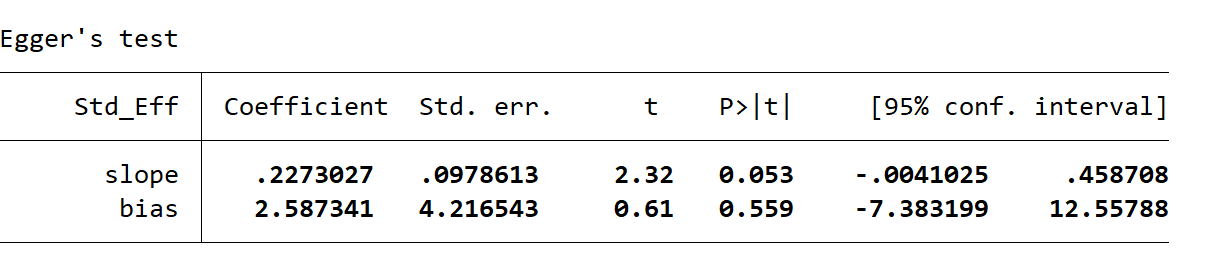


**Supplementary Figure 7.** Egger's test for Main Outcome
